# Biomaterials and Oxygen Join Forces to Shape the Immune Response and Boost COVID‐19 Vaccines

**DOI:** 10.1002/advs.202100316

**Published:** 2021-07-20

**Authors:** Thibault Colombani, Loek J. Eggermont, Zachary J. Rogers, Lindsay G. A. McKay, Laura E. Avena, Rebecca I. Johnson, Nadia Storm, Anthony Griffiths, Sidi A. Bencherif

**Affiliations:** ^1^ Department of Chemical Engineering Northeastern University Boston MA 02115 USA; ^2^ Department of Microbiology and National Emerging Infectious Diseases Laboratories Boston University School of Medicine Boston MA 02118 USA; ^3^ Department of Bioengineering Northeastern University Boston MA 02115 USA; ^4^ Harvard John A. Paulson School of Engineering and Applied Sciences Harvard University Cambridge MA 02138 USA; ^5^ Biomechanics and Bioengineering (BMBI) UTC CNRS UMR 7338 University of Technology of Compiègne Sorbonne University Compiègne 60203 France

**Keywords:** COVID‐19, injectable cryogel, neutralizing antibodies, oxygen, severe acute respiratory syndrome coronavirus 2 (SARS‐CoV‐2) vaccine

## Abstract

Severe acute respiratory syndrome coronavirus 2 (SARS‐CoV‐2) has led to an unprecedented global health crisis, resulting in a critical need for effective vaccines that generate protective antibodies. Protein subunit vaccines represent a promising approach but often lack the immunogenicity required for strong immune stimulation. To overcome this challenge, it is first demonstrated that advanced biomaterials can be leveraged to boost the effectiveness of SARS‐CoV‐2 protein subunit vaccines. Additionally, it is reported that oxygen is a powerful immunological co‐adjuvant and has an ability to further potentiate vaccine potency. In preclinical studies, mice immunized with an oxygen‐generating coronavirus disease 2019 (COVID‐19) cryogel‐based vaccine (O_2_‐Cryogel_VAX_) exhibit a robust Th1 and Th2 immune response, leading to a sustained production of highly effective neutralizing antibodies against the virus. Even with a single immunization, O_2_‐Cryogel_VAX_ achieves high antibody titers within 21 days, and both binding and neutralizing antibody levels are further increased after a second dose. Engineering a potent vaccine system that generates sufficient neutralizing antibodies after one dose is a preferred strategy amid vaccine shortage. The data suggest that this platform is a promising technology to reinforce vaccine‐driven immunostimulation and is applicable to current and emerging infectious diseases.

## Graphical abstract

Cryogels loaded with severe acute respiratory syndrome coronavirus 2 (SARS‐CoV‐2) nucleocapsid (N) and receptor‐binding domain (RBD) proteins (antigens), CpG (adjuvant), and GM‐CSF (immunomodulatory factor) activate both arms of the adaptive immune system. Additionally, oxygen, used as a co‐adjuvant, boosts the production of binding/neutralizing antibodies against the virus. This advanced biomaterial offers a new avenue to reinforce vaccines against infectious diseases.

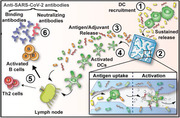

## 1 Introduction

Severe acute respiratory syndrome coronavirus 2 (SARS‐CoV‐2) has caused a global pandemic with over 30 million cases and nearly 1 million deaths as of September 2020 with no indications of slowing down.^[^ ^1^ ^]^ In response, several strategies are currently under rapid investigation, including treatments (e.g., antivirals, antibodies, anti‐inflammatory, and immunomodulatory factors),^[^ ^2^, ^3^, ^4^ ^]^ and prophylactic vaccines (e.g., nucleic acid‐based, protein subunit‐based, recombinant viral vector‐based, inactive or attenuated viral‐based, virus‐like particles).^[^ ^5^, ^6^, ^7^ ^]^ Yet, only vaccines have the potential to confer global immunity. The stakes are high: The alternative is natural herd immunity, requiring several waves of infection over the next few years, a period characterized by high mortality, economic uncertainty, and a perturbed way of life.^[^ ^8^ ^]^ Although two vaccine candidates have been initially approved by the Food and Drug Administration (FDA),^[^ ^9^, ^10^ ^]^ it is unclear if they will alter the course of the pandemic and confer long term immunity. Therefore, there is a critical need to continue driving novel vaccination platforms into the clinic until SARS‐CoV‐2 is quelled, as well as to prepare for future pandemics.

Protein subunit vaccines have been approved to protect against infectious diseases with several currently commercially available,^[^ ^11^ ^]^ yet they often lack the immunogenicity required to induce strong and long‐lasting immunity.^[^ ^5^, ^12^ ^]^ This is also exemplified by the recent delay of the Sanofi/GSK adjuvanted recombinant protein‐based coronavirus disease 2019 (COVID‐19) vaccine that demonstrated insufficient responses in older adults.^[^ ^13^ ^]^ Biomaterial‐based delivery systems can address this challenge^[^ ^14^ ^]^ by enhancing vaccine immunogenicity while reducing toxicity through controlled presentation and release of antigens and immunomodulatory factors (e.g., cytokines and adjuvants).^[^ ^15^ ^]^ Cryogels, polymeric biomaterials with a unique interconnected macroporous network, can be used as a platform for the controlled delivery of vaccine components, as well as, to recruit, host, and program immune cells in situ. Previously, cryogels have been leveraged for cancer vaccines with promising results in preclinical melanoma and breast cancer models.^[^ ^16^, ^17^ ^]^ Therefore, we hypothesized that a COVID‐19 cryogel‐based vaccine (Cryogel_VAX_), consisting of immunomodulatory factors and viral antigens, would provide an effective platform to promote the activation of dendritic cells (DCs), master regulators of the immune response, and stimulate antibody‐producing B cells in the draining lymph nodes (LNs). This strategy is expected to induce high titers of binding and neutralizing antibodies, resulting in an effective protection against SARS‐CoV‐2 infection.

Hypoxia, a hallmark of inflamed, infected, or damaged tissues, is due to an imbalance between oxygen supply and consumption. Additionally, this state is also inherent to biomaterials when injected or implanted into the subcutaneous space, likely due to poor vascularization and insufficient oxygen supply.^[^ ^18^ ^]^ A low oxygen tension has shown to negatively impact DC function, including survival, differentiation, migration, activation, and antigen presentation.^[^ ^19^, ^20^, ^21^ ^]^ Ultimately, this condition represents a major challenge when priming DCs, including with protein subunit vaccines. As a result, we proposed that oxygen supply from the cryogel platform could reverse local hypoxia and potentiate the immune responses induced by SARS‐CoV‐2 protein subunit vaccines.

In this study, we fabricated SARS‐CoV‐2 vaccines by incorporating both the nucleocapsid (N) protein and the receptor‐binding domain (RBD) of the spike (S) protein into hyaluronic acid‐based cryogels. The RBD protein is responsible for virus entry into cells and induces the production of high affinity antibodies.^[^ ^22^ ^]^ The N protein, known to be highly immunogenic, encapsulates the viral genomic RNA and contains specific T cell epitopes.^[^ ^23^, ^24^ ^]^ Combining these two proteins may induce a strong multi‐epitope immune response, activating both arms of the adaptive immune system (cell‐based and humoral), and ultimately leading to the production of antibodies with high neutralizing activity. The cryogels also contained granulocyte macrophage colony‐stimulating factor (GM‐CSF), a molecule that stimulates various immune cells, including DCs,^[^ ^25^, ^26^ ^]^ and the adjuvant CpG ODN 1826, a ligand for TLR9 (toll‐like receptor 9) that activates DCs, specifically plasmacytoid DCs.^[^ ^27^, ^28^, ^29^ ^]^ These cryogel‐based vaccines were formulated to induce a robust humoral immune response.^[^ ^29^, ^30^ ^]^ To enhance vaccine immunogenicity, oxygen was considered as an immunological co‐adjuvant that would eliminate local hypoxia at the site of vaccine administration.^[^ ^31^ ^]^ Thus, oxygen‐producing calcium peroxide (CaO_2_) particles and acrylate‐PEG‐catalase (APC) were incorporated within the cryogel‐based vaccine formulations before freezing, as previously described.^[^ ^32^ ^]^ The resulting oxygen‐generating COVID‐19 cryogel‐based vaccine (O_2_‐Cryogel_VAX_) was designed to generate oxygen upon the reaction of CaO_2_ with water and to eliminate hydrogen peroxide byproducts through a catalase‐mediated breakdown. Herein, we first characterized the encapsulation and release of biomolecules from O_2_‐Cryogel_VAX_ and their potential to reverse hypoxia‐associated DC inhibition. Next, we investigated the ability of the vaccines to promote immune cell infiltration locally, stimulate B cell expansion, and induce high antibody titers with strong neutralizing activity against the virus. Finally, we evaluated vaccine‐induced antibody subclasses, T helper responses, cytokine secretion, and the resulting balance between Th1 and Th2 immune responses.

## 2 Results

### 2.1 Severe Acute Respiratory Syndrome Coronavirus 2 Vaccine Rational, Fabrication, and Characterization

**Figure 1:**
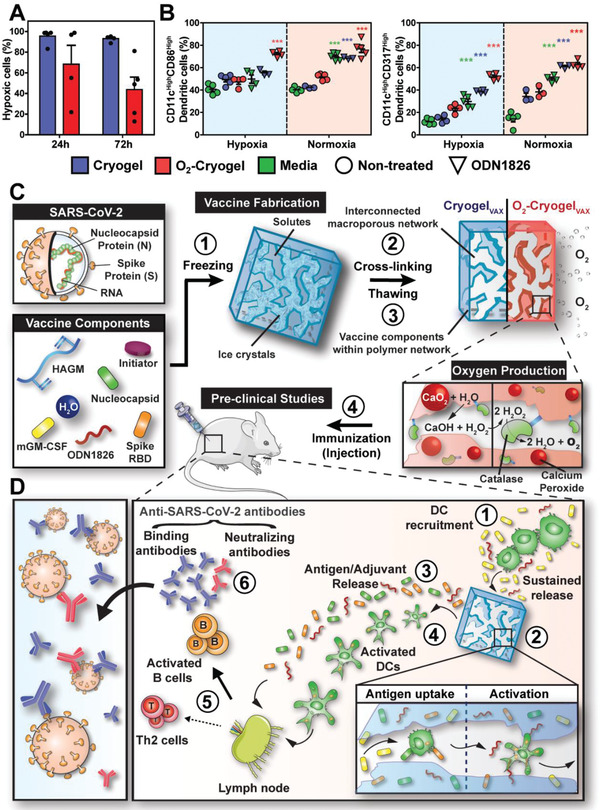
Cryogel‐based vaccines reinforce DC‐mediated immune responses. A) Quantification of cellular hypoxia of infiltrated cells within cryogels and O_2_‐cryogels at 24 and 72 h following post‐subcutaneous injection in mice with Hypoxyprobe‐1. B) Quantitative flow cytometric measurements of CD86^High^ and CD317^High^ DCs (CD11c^High^) in cryogels or O_2_‐cryogels in the presence or absence of CpG ODN 1826 in hypoxia and normoxia for 24 h (right panel). C) Overview of the process for fabrication and evaluation of square‐shaped COVID‐19 cryogel‐based (Cryogel_VAX_) and O_2_‐cryogel‐based (O_2_‐Cryogel_VAX_) vaccines. Step 1 involves freezing vaccine components, enabling crosslinking of solutes around ice crystals (step 2). Thawing results in an interconnected macroporous network with vaccine components encapsulated within the polymer network (step 3). Addition of calcium peroxide and catalase to the vaccine components before cryogelation produces O_2_‐Cryogel_VAX_ capable of sustained production of oxygen. In step 4, cryogels are subcutaneously injected into mice for preclinical vaccine studies. D) Illustration describing a model for DC‐enhanced cryogel‐induced immunity. Initiator system: APS and TEMED. Values represent the mean ± SEM (*n* = 5). Data were analyzed using two‐way ANOVA and Bonferroni post‐tests to evaluate differences between conditions (colored stars indicate statistical significance within a given group of the same color), ***p < 0.001. Mouse carton taken from smart.servier.com.

The capacity of O_2_‐cryogels to reduce local hypoxia was first evaluated. O_2_‐cryogels and cryogels were fabricated as previously described,^[^ ^32^ ^]^ and their ability to generate oxygen in vitro was confirmed using contactless environmental oxygen probes (Figure S1, Supporting Information). As intended, O_2_‐cryogels released oxygen in their surrounding environment for nearly 48 h, with an oxygen level peaking at over 310 µM after 3 h. On the contrary, catalase‐free O_2_‐cryogels produced an insignificant amount of oxygen, confirming the need to use this enzyme. Next, O_2_‐cryogels and cryogels were subcutaneously injected in mice, and in vivo cellular hypoxia was tested with Hypoxyprobe‐1 after 24 and 72 h (**Figure** **1**A). As expected, 95 ± 3% of infiltrated cells within (non‐oxygen producing) cryogels were hypoxic as early as 24 h following injection. However, the fraction of hypoxic cells was significantly decreased for O_2_‐cryogels to 68 ± 18% and 43 ± 12% after 24 and 72 h, respectively. We also assessed the capacity of the biomaterials to prevent the inhibition of DCs when subjected to hypoxia. In vitro, bone marrow‐derived dendritic cells (BMDCs) were stimulated with CpG ODN 1826 in normoxic or hypoxic conditions for 24 h and exposed to either cryogels or O_2_‐cryogels. As shown in Figure 1B, O_2_‐cryogels restored DC activation with increased fractions of CD86^High^ and CD317^High^ DCs, comparable to DCs stimulated under normoxic conditions. This suggests that local oxygen supply from cryogel‐based vaccines could potentiate DC activation and enhance SARS‐CoV‐2 protein subunit vaccines. Therefore, O_2_‐cryogels were used in this study as a biomaterial of choice to improve protein‐based SARS‐CoV‐2 vaccines.

Next, hyaluronic acid‐based cryogel vaccines were fabricated by cryogelation, as previously described (Figure 1C, steps 1–3).^[^ ^16^, ^26^, ^33^ ^]^ This process results in an elastic construct with a highly interconnected macroporous network, allowing immune cells to traffic in and out of the cryogel. The encapsulation of RBD and N proteins within O_2_‐Cryogel_VAX_ polymer walls was characterized by confocal microscopy and their release from the cryogel by enzyme‐linked immunosorbent assay (ELISA) (Figure S2A,B, Supporting Information). The two proteins were effectively entrapped within the polymer network. Furthermore, both cryogel‐based vaccines (Cryogel_VAX_ and O_2_‐Cryogel_VAX_) led to an initial burst release of the immunomodulatory factors (GM‐CSF and CpG ODN 1826) and RBD antigen within the first 8 h and then about 35% of CpG ODN 1826, 91% of GM‐CSF, and 71% of RBD were delivered by 30 h (experimental endpoint) (Figure S2B, Supporting Information). Notably, there were no significant differences between the encapsulation (Figure S2C, Supporting Information) and release profiles of CpG ODN 1826, GM‐CSF, and RBD for the two types of cryogels. Importantly, negligible amounts of Ca^2+^ and H_2_O_2_ were released from O_2_‐cryogels (Figure S2D,E, Supporting Information), resulting in high BMDC viability (Figure S2F, Supporting Information). These results suggested that cryogels and O_2_‐cryogels are suitable platforms for controlled vaccine delivery.

We hypothesized that the protein antigens and adjuvants released from the vaccine would most likely be drained to the inguinal LNs and direct B cell activation.^[^ ^34^, ^35^, ^36^ ^]^ Additionally, following immunization (Figure 1C), we anticipated that the cryogel‐based vaccines would induce DC‐mediated humoral immunity (Figure 1D).^[^ ^26^ ^]^ This is supported with our previous findings on cryogel cancer vaccines where a sustained release of immunomodulatory factors (GM‐CSF and CpG ODN 1826) promoted DC infiltration and stimulation.^[^ ^26^, ^32^, ^33^ ^]^ Within the cryogels, DCs are expected to take up N and RBD protein antigens and be simultaneously stimulated by CpG ODN 1826 and additive oxygen. Activated, antigen‐loaded DCs would then migrate to the draining LNs, activate antigen‐specific T cells, and ultimately boost antibody‐producing B cells. A subset of activated B cells, differentiating into plasma cells, would produce large quantities of SARS‐CoV‐2‐binding antibodies. A fraction of these antibodies known as neutralizing antibodies would exert their inhibitory activity by abrogating binding of the virus RBD to the human receptor angiotensin‐converting enzyme 2 (ACE2).

### 2.2 Oxygen‐Generating COVID‐19 Cryogel‐Based Vaccine and Cryogel‐Based Vaccine Induce High Antibody Titers with Strong Neutralizing Activity

**Figure 2:**
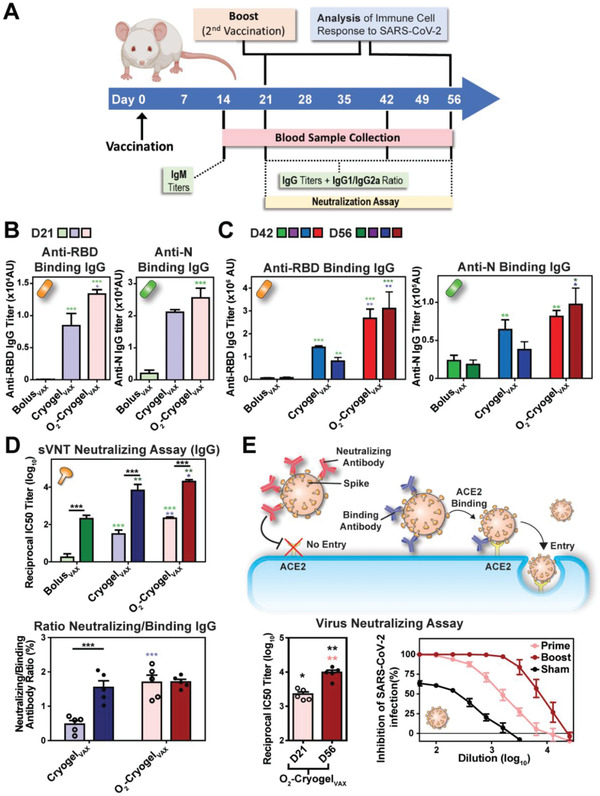
O_2_‐Cryogel_VAX_ induces robust binding and neutralizing antibody responses against SARS‐CoV‐2 in mice. A) Study timeline describing the vaccination regimen (BALB/c mice; *n* = 5 per group) and the timing of the different sample collection and immunoassay performed in this study. B) Post‐prime endpoint titers of RBD and N‐specific IgG antibody determined by ELISA at day 21. C) Post‐boost endpoint titers of RBD and N‐specific IgG antibody determined by ELISA at day 42 and 56. D) SARS‐CoV‐2 surrogate virus neutralization test (sVNT) and binding/neutralizing antibody ratio at day 21 and 56. E) Virus neutralization assay (i.e., PRNT) using VeroE6 cells infected with authentic SARS‐CoV‐2. Neutralizing antibodies from O_2_‐Cryogel_VAX_‐treated mice were tested after prime (day 21: D21) and prime‐boost (day 56: D56) immunizations. Values are representative of individual serum sample and are shown as mean ± SEM (*n* = 5–10). Data were analyzed using one‐way ANOVA and Bonferroni post‐tests to evaluate differences between time points (underlined dark stars indicate statistical significance) or two‐way ANOVA and Bonferroni post‐tests to evaluate differences between different conditions/treatments at the same time point (colored stars indicate statistical significance within a given condition of the same color), *p < 0.05, **p < 0.01, and ***p < 0.001.

To test the vaccines, 8‐week‐old female BALB/c mice were immunized by subcutaneous injection of two O_2_‐Cryogel_VAX_ or Cryogel_VAX_ (one on each flank) at day 0 (prime) and day 21 (boost) (**Figure ** **2**A). Control groups were injected with either phosphate buffered saline (PBS) (sham–negative control), cryogel‐free vaccine (Bolus_VAX_), or Freund's‐based vaccine (Freund_VAX_–positive control) **Table** **1**. Blood serum analysis revealed that, although low titers of immunoglobulin M (IgM) antibodies were found across all groups (Figure S3A, Supporting Information), Cryogel_VAX_ and O_2_‐Cryogel_VAX_ induced high titers of RBD‐specific binding immunoglobulin G (IgG) antibodies after only 21 days (Figure 2B, Figure S3B, Supporting Information). These titers increased substantially following boost immunization, peaking at 1.4×10^6^ at day 42 for animals immunized with Cryogel_VAX_ and 3.1 x 10^6^ at day 56 for animals immunized with O_2_‐Cryogel_VAX_, amounts two orders of magnitude greater than those in the control groups (Figure 2C, Figure S3B, Supporting Information). Interestingly, O_2_‐Cryogel_VAX_ induced higher production of RBD‐specific binding IgG antibodies than Cryogel_VAX_ did, showing a fivefold increase at day 56, and these titers were sustained for nearly 2 months (study endpoint). Similarly, immunization with O_2_‐Cryogel_VAX_ resulted in high titers of N‐specific binding IgG antibodies comparable to those induced by Freund_VAX_, and 3 and 5 times higher than those generated by Cryogel_VAX_ and Bolus_VAX,_ respectively.

**Table 1:** SARS‐CoV‐2 vaccination groups and dosage

Group	Vaccine formulation
Sham	2 × 100 µL PBS
Freund_VAX_	1 × 100 µL [(25 µg RBD + 25 µg N + 1.5 µg GM‐CSF—1:1 ratio with CFA (Prime) or IFA (Boost)]
Bolus_VAX_	2 x [(10 µg RBD + 10 µg N + 1.5 µg GM‐CSF + 50 µg CpG ODN 1826) + 100 µL PBS]
Cryogel_VAX_	2 x [(10 µg RBD + 10 µg N + 1.5 µg GM‐CSF + 50 µg CpG ODN 1826) + 100 µL PBS]
O_2_‐Cryogel_VAX_	2 x [(10 µg RBD + 10 µg N + 1.5 µg GM‐CSF + 50 µg CpG ODN 1826 + 200 µg of APC + 200 µg CaO_2_) + 100 µL PBS]

To detect neutralizing antibodies that target the viral spike (S) protein RBD and block its interaction with ACE2, we performed a SARS‐CoV‐2 surrogate virus neutralization test (sVNT) (Figure 2D, Figure S3C, Supporting Information). In agreement with the high serological IgG titers, O_2_‐Cryogel_VAX_ elicited the strongest neutralizing antibody response, with a reciprocal IC_50_ titer of nearly 20000 at day 56, which is 3 and 100‐fold higher than those from Cryogel_VAX_ and control groups (Bolus_VAX_ and Freund_VAX_), respectively. Additionally, neutralizing antibodies induced within 3 weeks after only a single immunization with O_2_‐Cryogel_VAX_ were comparable to those induced after 8 weeks in mice receiving prime and boost vaccinations with Bolus_VAX_ or Freund_VAX_ (Figure 2D upper). Importantly, 1.7% of O_2_‐Cryogel_VAX_‐induced anti‐RBD IgG antibodies were neutralizing from day 21 onward (Figure 2D lower). We also assessed the neutralization potency of antibodies by plaque reduction neutralization test (PRNT) using VeroE6 cells infected with authentic SARS‐CoV‐2 (Figure 2E).^[^ ^34^ ^]^ As expected, O_2_‐Cryogel_VAX_ immunization led to high neutralizing titers, which intensified from day 21, reaching a reciprocal IC_50_ value of nearly 10 000 at day 56 (study endpoint). Collectively, these data demonstrated that the cryogel platform potentiates vaccine efficacy. Furthermore, additive oxygen as a co‐adjuvant strongly boosted the humoral response, as shown by the production of antibodies with high neutralizing activity and presumably increased binding affinity to RBD.

### 2.3 Oxygen‐Generating COVID‐19 Cryogel‐Based Vaccine Promotes Local Immune Cell Recruitment and B Cell Expansion in Lymph Nodes

**Figure 3:**
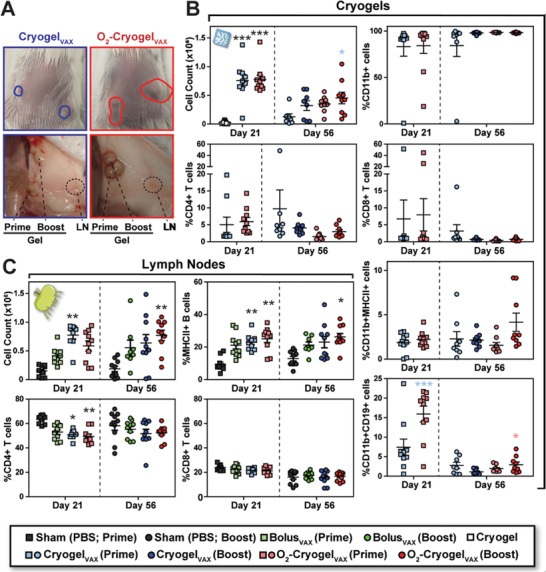
O_2_‐Cryogel_VAX_ recruits high number of CD19+ leukocytes and stimulates B cell expansion in the LNs. A) Photographs of cryogels and LNs at day 56 following subcutaneous injection. The plain circles depict the locations of Cryogel_VAX_ (blue) and O_2_‐Cryogel_VAX_ (red) under the skin. Immune cell populations in B) cryogels and C) LNs as analyzed by flow cytometry. Two draining LNs and cryogels were analyzed per animal. Values, indicative of individual LNs or cryogels, are presented as mean ± SEM (*n* = 10). Data were analyzed using two‐way ANOVA and Bonferroni post‐tests to evaluate differences between conditions at the same time point (colored stars indicate statistical significance within a given condition of the same color), *p < 0.05, **p < 0.01, and ***p < 0.001.

To understand how the vaccines work, we characterized the immune response following prime and prime‐boost immunizations in mice. At day 21 and 56, draining LNs, spleens, and cryogels were explanted (Figure 2A). In comparison to the injection sites of Cryogel_VAX_, sites of both prime and boost O_2_‐Cryogel_VAX_ injections were markedly enlarged, indicating increased inflammation and immune cell infiltration (**Figure** **3**A). Nonetheless, no rash or stress was observed in mice, suggesting that the vaccines were well tolerated. Overall, unlike blank cryogels, large numbers of infiltrated immune cells were retrieved from both types of cryogel‐based vaccines (Figure 3B, Figure S4A,B, Supporting Information). Most explanted cryogels exhibited low and comparable numbers of CD4+ and CD8+ T cells, whereas high numbers of CD11b‐positive myeloid cells, but no DCs, were present (Figure 3B). Furthermore, the total number of cells positive for the B cell marker CD19 was twofold higher in O_2_‐Cryogel_VAX_ compared to those in blank cryogels (Figure S4B, Supporting Information). However, the exact identity of these cells is unclear, as they were also CD11b‐positive and did not have other B cell markers such as MHCII (Figure S4A, Supporting Information). Additionally, only a small population of MHCII‐positive CD11b+ cells was observed. Interestingly, evidence for an ongoing adaptive immune response was found in a small number of cryogel‐based vaccines. These cryogel‐based vaccines contained a lower fraction of CD11b+ myeloid cells but relatively greater proportions of T cells and MHCII+ B cells (see outliers in Figure 3B, Figure S4B, Supporting Information). Compared to day 21, immune cell numbers in prime cryogel‐based vaccines decreased at day 56, indicating that both types of cryogel‐based vaccines do not generate chronic and potentially dangerous inflammatory responses.

Analysis of LNs in mice immunized with Cryogel_VAX_ and O_2_‐Cryogel_VAX_ confirmed that a robust immune response was induced. This resulted in at least a fourfold greater increase in total immune cell numbers than that observed among mice receiving sham injections at both time points (Figure 3C, Figure S5A,B, Supporting Information). In particular, the frequency of MHCII+ B cells within LNs was considerably increased in mice immunized with both cryogel‐based vaccines. Although the frequency of CD4+ T cells was reduced at day 21 in LNs from mice receiving cryogel‐based vaccines (Figure 3C), overall CD4+ and CD8+ T cell numbers increased after vaccination (Figure S5B, Supporting Information). These data showed that cryogel‐based vaccines induce a strong B cell‐mediated immune response in LNs and display restrained adaptive immune responses within the cryogels following initial priming.

### 2.4 Oxygen‐Generating COVID‐19 Cryogel‐Based Vaccine Enhances Both Th1‐ and Th2‐Associated Immune Responses

**Figure 4:**
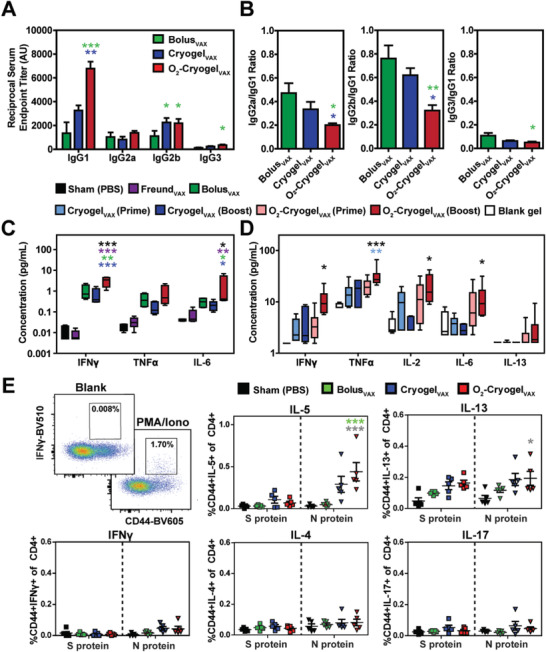
Immunization with O_2_‐Cryogel_VAX_ triggers a balanced Th1/Th2‐mediated immune response against SARS‐CoV‐2. A) Endpoint titers and B) endpoint titer ratios of the different IgG subclasses after 56 days were assessed by ELISA. Th1 and Th2 cytokine levels were measured in mouse serum at C) day 24 and in explanted cryogels at D) day 56 by multiplex assay. E) Flow cytometry gating and frequencies of cytokine‐producing CD44+CD4+ T cells following S and N protein‐derived peptide stimulation of splenocytes isolated at day 21. Data are represented as A–D) mean ± SEM (*n* = 5) or E) values of individual spleens (*n* = 5). Data were analyzed using two‐way ANOVA and Bonferroni post‐tests to evaluate differences between groups (colored stars indicate statistical significance within a given condition of the same color), *p < 0.05, **p < 0.01, and ***p < 0.001.

Next, to evaluate the immunostimulatory effects of cryogel‐based SARS‐CoV‐2 vaccines, we analyzed the balance between Th1 and Th2 immune responses. Production of antibody subclass IgG1 is indicative of Th2 responses, and IgG2a/b/c and IgG3 are indicative of Th1 responses.^[^ ^35^ ^]^ In our study, vaccines across all groups elicited IgG2 and IgG1 subclass RBD‐binding antibodies, indicating induction of both Th1 and Th2 immune responses (**Figure** **4**A). Both cryogel‐based vaccines promoted the production of IgG2b. However, O_2_‐Cryogel_VAX_ improved IgG1 production resulting in lower IgG2a/IgG1 and IgG2b/IgG1 ratios (Figure 4B). Interestingly, O_2_‐Cryogel_VAX_ was the only vaccine that induced IgG3 production (Figure 4A,B). Importantly, relatively low IgE titers were detected for Bolus_VAX_, Cryogel_VAX_, and O_2_‐Cryogel_VAX_ (Figure S6A, Supporting Information), suggesting that mice did not develop an allergic reaction to the vaccines.

Th1 and Th2 responses are also associated with different cytokine profiles: Interferon *γ* (IFN*γ*), tumor necrosis factor *α* (TNF*α*), and interleukin‐2 (IL‐2) indicate Th1 responses; IL‐4, IL‐5, and IL‐13 indicate Th2 responses. Thus, we quantified cytokines in serum at day 24 (Figure 4C, Figure S6B, Supporting Information) and in explanted cryogels at day 56 (Figure 4D). At day 24, all vaccines induced detectable concentrations of pro‐inflammatory interleukin‐6 (IL‐6) and Th1 cytokines (IFN*γ* and TNF*α*) in mouse sera (Figure 4C). Interestingly, concentrations of IFN*γ* and IL‐6 in mice immunized with O_2_‐Cryogel_VAX_ were threefold or tenfold higher than their concentrations in mice immunized with Bolus_VAX_ or Cryogel_VAX_, respectively. Additionally, low levels of IL‐4 were detected with no increased secretion of IL‐5 and IL‐13 (Figure S6B, Supporting Information). Similar results were observed at day 56 (Figure 4D). Higher concentrations of Th1 cytokines IFN*γ*, TNF*α*, and IL‐2, as well as, IL‐6, were quantified in mice immunized with O_2_‐Cryogel_VAX_, compared to those immunized with Cryogel_VAX_. Furthermore, we noted low concentrations of the Th2 cytokine IL‐13 in O_2_‐Cryogel_VAX_. As expected, blank cryogels were associated with low or negligible amounts of these cytokines.

To more directly assess the Th1/Th2 immune responses, we investigated the cytokine profile of antigen‐specific T cells generated with both cryogel‐based vaccines. The intracellular production of cytokines by splenocytes from immunized mice was examined following stimulation with peptides derived from viral S or N proteins. Cells were isolated at day 21 after prime immunization. Splenocytes from O_2_‐Cryogel_VAX_‐immunized mice stimulated with N‐derived peptides showed increased fractions of IL‐5‐producing CD4+ and CD8+ T cells and IL‐13‐producing CD4+ T cells (Figure 4E, Figure S7, Supporting Information). These results indicated the presence of N protein‐specific Th2 cells. However, no differences were noted following stimulation with S‐derived peptides, and the proportions of IFN*γ*, IL‐4, or IL‐17‐producing T cells were also comparably low. Collectively, these data suggested that both types of cryogel‐based vaccines elicited balanced Th1/Th2 immune responses, even though it was more prominent for O_2_‐Cryogel_VAX_.

## 3 Discussion

Nearly every decade for the past 30 years, a novel coronavirus pandemic emerges, pushing the healthcare system to its limit.^[^ ^36^ ^]^ Although the current outbreak had long been predicted, SARS‐CoV‐2 has created the most severe crisis in recent history.^[^ ^37^, ^38^ ^]^ The rapid development of an effective and safe vaccine against this virus is the most effective strategy to end this pandemic. Among them, protein subunit vaccines have been widely investigated against SARS‐CoV‐2 due to their performance and safety record, and such vaccines have already shown promising early results in phase 1/2 clinical trials.^[^ ^14^ ^]^ Yet, subunit vaccines still have to overcome their lack of immunogenicity.^[^ ^39^ ^]^ In addition, an ideal antiviral vaccine should be versatile and rapid to design, enabling rapid response to the public health emergency. To overcome these challenges, our team leveraged a cryogel‐based vaccine platform to strengthen protein subunit vaccines and induce a strong and sustained immunity against SARS‐CoV‐2.^[^ ^26^ ^]^ In addition, we showed that oxygen is a powerful immunological co‐adjuvant that shapes and reinforces the immune response. This work demonstrated how robust and modular the cryogel‐based vaccine technology is, which was successfully and quickly adapted from cancer to an infectious disease at breakneck speed (< 3 months).

We found that Cryogel_VAX_ triggers both Th1 and Th2 immune responses while enhancing the efficacy of a conventional protein subunit vaccine by 100‐fold (Bolus_VAX_). This is most likely due to the ability of cryogels to control the release of immunomodulatory factors to stimulate B cells in the draining LNs while activating high numbers of immune cells within the cryogels. Following prime‐boost immunization, Cryogel_VAX_ elicited a strong humoral immune response for nearly 2 months (study endpoint) and was associated with high levels of anti‐RBD IgG antibodies and strong neutralizing activity to SARS‐CoV‐2. In addition, Cryogel_VAX_ induced CD4+ and CD8+ T cell responses, specifically directed against the N protein. The unique macroporous architecture of Cryogel_VAX_ and incorporation of a chemoattractant also promoted the recruitment of resident leukocytes and CD19+ immune cells, which likely increased B cell expansion in the LNs within 21 days. These data are in agreement with our previous work demonstrating that immune cells can traffic in and out of the cryogels.^[^ ^26^ ^]^ Furthermore, this supports our hypothesis that Cryogel_VAX_ may act as a distant immune cell training platform that would reinforce our prime‐boost vaccination strategy.

Although promising, Cryogel_VAX_ has been associated with a number of limitations in this study, including a decrease in anti‐SARS‐CoV‐2 IgG antibodies after 42 days and low concentrations of Th1 cytokines. In light of these findings, we explored the use of oxygen as an immunological co‐adjuvant to potentiate vaccine efficacy. We confirmed that O_2_‐cryogels did not produce any harmful byproducts and were cytocompatible while generating a controlled level of oxygen. We demonstrated that supplemental oxygen not only promoted CD4+ and CD8+ T cell responses against SARS‐CoV‐2, but also promoted the production of Th1‐biased and pro‐inflammatory cytokines. Additionally, O_2_‐Cryogel_VAX_ remarkably boosted humoral immunity with long‐lasting production of binding antibodies (fivefold higher at day 56) and high IgG1 neutralizing activity with a single injection. This suggests the induction of a balanced Th1 and Th2‐associated immune responses. Strikingly, IgG1 skewing was observed with O_2_‐Cryogel_VAX_. This may be attributed to oxygen transport from the vaccine site to the germinal centers, presumably leading to IgG1 class switching and proliferation of activated B cells when exposed to increased oxygen tension.^[^ ^43^ ^]^ Interestingly, O_2_‐Cryogel_VAX_ promoted local recruitment of leukocytes, notably CD19+ cells, and enhancement of B cell expansion in the LNs. Furthermore, no vaccine‐driven allergic inflammation was observed as supported by the low or negligible levels of serum IgE antibodies and cytokines involved in allergic reactions (IL‐4, IL‐5, and IL‐13). Altogether, this study highlights that oxygen could become a key co‐adjuvant in vaccine development and play an important role in shaping the immune response, ultimately boosting vaccine efficacy.

More research is needed to assess the duration of our vaccine‐induced immune responses, especially in non‐human primates and humans, as well as to more carefully dissect the immune mechanism by which both the humoral and cellular immune responses are triggered.^[^ ^40^, ^41^ ^]^ Furthermore, examining immune cell populations at earlier time points, the synergistic interaction of N and RBD proteins during immune priming, and the contribution of each of the immunomodulatory factors may further shed light on the vaccine mode of action. Although we previously reported that CpG ODN 1826 and GM‐CSF can be released up to 40 days from cryogels,^[^ ^26^ ^]^ the release kinetics of the antigens and adjuvant from O_2_‐Cryogel_VAX_ need to be further investigated and over an extended period of time. Moreover, a deeper understanding of the spatiotemporal diffusion of oxygen could be leveraged to further boost vaccine efficacy. While cryogel‐based vaccines have proven to induce long‐lasting immunity against melanoma,^[^ ^26^ ^]^ additional studies are required to confirm long‐lived protective immunity and the induction of central and effector T cell memory in the context of COVID‐19. In addition, the effectiveness of O_2_‐Cryogel_VAX_ in aged or obese animals needs to be tested. Finally, a toxicology analysis in combination with a careful evaluation of biomaterial degradation are necessary to assess long‐standing vaccine safety_._

In summary, our study unveils the magnitude of an advanced biomaterial‐based technology to harness the power of protein subunit vaccines, leading to a rapid and protective anti‐SARS‐CoV‐2 immune response. Additionally, we report the synergistic effect of vaccines engineered to produce oxygen as a powerful immunological co‐adjuvant. Finally, although our efforts focused on protein subunits, this platform is compatible with other strategies, such as live attenuated or inactivated pathogens and nucleic acid vaccines, and may boost the efficiency of existing vaccines or those under development. While several vaccines have proven to be effective in phase 3 clinical trials, their stability as well as the number of required doses and the duration of their protection might not be optimal.^[^ ^42^ ^]^ Our study opens new possibilities to leverage vaccines, such as the one described here against COVID‐19, and help quickly develop new versions as the virus could mutate and be more infectious. For instance, the recent new viral mutations of SARS‐CoV‐2 leading to various virus variants around the globe appear to be more contagious and spread more easily. This is a good example of the threat and danger of SARS‐CoV‐2 rapid mutability, leading to more death, economic uncertainty, and travel restrictions.^[^ ^43^, ^44^ ^]^ Finally, this platform is potentially applicable in reinforcing vaccines for other infectious diseases and conditions that may require boosting the immune system.

## 4 Experimental Section

### Cryogel Fabrication

Cryogels were fabricated as previously described by redox‐induced free radical cryopolymerization of hyaluronic acid glycidyl methacrylate (HAGM, 4% w/v) at subzero temperature (−20 °C).^[^ ^16^, ^26^, ^32^ ^]^ Briefly, the polymer solution was precooled at 4 °C prior to adding tetramethylethylenediamine (TEMED, 0.42% w/v, Sigma‐Aldrich) and ammonium persulfate (APS, 0.84% w/v, Sigma‐Aldrich). Then, the mixture was transferred into Teflon molds (4 mm × 4 mm × 1 mm, cubiform with 2 square‐shaped sides), placed in a freezer at −20 °C, and allowed to cryopolymerize for 16 h. Finally, the newly formed cryogels were thawed at room temperature (RT) to remove ice crystals and washed with Dulbecco's PBS (Gibco). For O_2_‐cryogel fabrication, APC (1% w/v, Sigma‐Aldrich), and CaO_2_ (1% w/v) were mixed with the cryogel polymer solution before the addition of TEMED and APS as previously reported.^[^ ^32^ ^]^

### Severe Acute Respiratory Syndrome Coronavirus 2 Vaccine Fabrication

Protein subunit‐based vaccines were fabricated by formulating purified recombinant SARS‐CoV‐2 Spike (ΔTM) his‐tagged protein (RBD, 10 YP_0 097 24390.1—Arg319‐Phe541, Creative Biomart nCoVS‐125V), purified recombinant 2019‐nCoV Nucleocapsid protein (N, YP_0 097 24397.2, Creative Biomart N‐127V), purified recombinant mouse GM‐CSF (GenScript), and synthetic immunostimulatory oligonucleotide containing unmethylated CpG dinucleotides (CpG ODN 1826, 5’‐tccatgacgttcctgacgtt‐3’, VacciGrade, InvivoGen) in PBS. For Bolus_VAX_, 10 µg RBD, 10 µg N, 1.5 µg GM‐CSF, and 50 µg CpG ODN 1826 were formulated in 100 µL of PBS. For Cryogel_VAX_ and O_2_‐Cryogel_VAX_, 10 µg RBD, 10 µg N, 1.5 µg GM‐CSF, and 50 µg CpG ODN 1826 (per gel) were incorporated within the polymer solution prior to cryogelation. After thawing, each cryogel‐based vaccine was resuspended in 100 µL of PBS. For Freund_VAX_ (positive control), 25 µg RBD, 25 µg N, and 3 µg GM‐CSF were formulated in 50 µL PBS and mixed at a 1:1 ratio with complete Freund's adjuvant (CFA—Prime) or incomplete Freund's adjuvant (IFA—Boost). Sham vaccine formulation containing only 100 µL PBS was used as a negative control.

### Oxygen Release Kinetics

Kinetics of oxygen release were determined using contactless optical oxygen microprobes (PyroScience GmbH, Aachen, Germany). Briefly, square‐shaped cryogels, O_2_‐cryogels, and APC‐free O_2_‐cryogels were individually placed into a 96 well plate containing 200 µL of PBS and incubated at 37 °C under normoxic conditions using a Napco CO_2_ 1000 incubator (Thermo Fisher Scientific). The microprobe was centered in the bottom of the wells, and dissolved oxygen (µmol L^−1^, 1 point every 300 s) within the surrounding environment of cryogels were recorded for 48 h.

### Mouse Model and Study Design

Animal experiments were carried out in compliance with the National Institutes of Health (NIH) guidelines and approved by the Division of Laboratory Animal Medicine and Northeastern University Institutional Animal Care and Use Committee (protocol number 20–0629R). Vaccination studies were performed on 6–8‐week‐old female BALB/c (Charles River). Freund_VAX_ was inoculated intraperitoneally (IP) (1 injection/mouse). Sham, Bolus_VAX_, Cryogel_VAX,_ and O_2_‐Cryogel_VAX_ were injected subcutaneously (SC) in both flanks (total of 2 injections/mouse). Boost injections were performed 21 days after priming at the same location. Blood samples were collected every 7 days from day 14 onward and three days post‐boost (day 24). Cryogel‐based vaccines, LNs, and spleens were harvested at day 21 (prime) and day 56 (prime + boost) and then dissociated as previously described.^[^ ^26^, ^32^ ^]^ Hypoxia studies were performed on 6–8‐week‐old female C57BL/6 mice (Charles River). Cryogels and O_2_‐cryogels were suspended in 100 µL PBS and injected SC into mouse flanks. After 23 or 71 h, mice were injected IP with 200 µL of Hypoxyprobe‐1 in PBS (dosage: 60 mg kg^−1^). Hypoxyprobe‐1 (i.e., Pimonidazole hydrochloride) is reductively activated in hypoxic cells, forming stable covalent adducts with thiol groups in proteins, peptides, and amino acids. Cellular hypoxia can then be detected by immunochemical means with the antibody reagent MAb1 that binds to these adducts. After 1 h of incubation, the cryogels and O_2_‐cryogels were harvested, dissociated, and stained with FITC‐MAb1 according to the manufacturer's recommendation. Fractions of hypoxic cells were then quantified by flow cytometry using an Attune NxT flow cytometer (Thermo Fisher Scientific).

### Bone Marrow‐Derived Dendritic Cells Isolation and Generation

DC activation studies were performed using BMDCs generated from 6–8‐week‐old female C57BL/6 mice (Charles River) as previously described.^[^ ^26^ ^]^ Briefly, femurs of mice were explanted, disinfected in 70% ethanol for 5 min, washed in PBS, and then bone ends were removed, and the marrow flushed with PBS (2 mL, 27G needle). Next, cells were mechanically dissociated by pipetting, centrifuged (5 min, 300 g), and resuspended (10^6^ cells mL^−1^) in Roswell Park Memorial Institute Medium (RPMI 1640, Gibco) supplemented with 10% heat‐inactivated fetal bovine serum (FBS, Sigma‐Aldrich), 100 U mL^−1^ penicillin (Gibco), 100 µg mL^−1^ streptomycin (Gibco), 2 × 10^–3^ m L‐glutamine (Gibco), and 50 × 10^–6^ m 2‐mercaptoethanol (Gibco). At day 0, BMDCs were seeded in non‐treated p6 well plates (2 × 10^6^ cells per well) in 5 mL of complete RPMI medium supplemented with 20 ng mL^−1^ GM‐CSF. At day 3, another 5 mL of RPMI medium containing 20 ng mL^−1^ GM‐CSF was added to each well. At day 6 and 8, half of the media was sampled from each well, centrifuged, and the cell pellet was resuspended in 5 mL of fresh RPMI media supplemented with only 10 ng mL^−1^ GM‐CSF before re‐plating. BMDCs were collected at day 10 (non‐adherent cells) and used to evaluate DC activation in normoxia or hypoxia.

### In Vitro Dendritic Cell Activation and Cytocompatibility Assay

BMDCs were incubated in complete RPMI medium containing 10 ng mL^−1^ GM‐CSF at 37 °C in either humidified 5% CO_2_/95% air (normoxic) or 5% CO_2_/1% O_2_/94% N_2_ (hypoxic) Napco CO_2_ 1000 incubator (Thermo Fisher Scientific) for 24 h. Cryogels, O_2_‐cryogels, or APC‐free O_2_‐cryogels were added to each well (1 cryogel/well) prior to incubation. For BMDC activation, the medium was supplemented with 5 µg mL^−1^ CpG ODN 1826. The negative control consisted of BMDCs cultured in complete RPMI medium containing 10 ng mL^−1^ GM‐CSF. DC stimulation and maturation were evaluated by flow cytometry using the following fluorescent antibodies (BioLegend): APC‐conjugated anti‐mouse CD11c (clone N418), PE‐conjugated anti‐mouse CD86 (Clone GL1), and PerCP/Cyanine5.5‐conjugated anti‐mouse CD317 (clone 927). DC viability was evaluated by flow cytometry using a Fixable Viability Dye eFluor 780 (eBioscience).

### Imaging of Encapsulated N and Receptor‐Binding Domain Proteins within the Cryogel Network

RBD or N protein was dissolved in sodium bicarbonate buffer (pH 8.5) at 0.5 mg mL^−1^ and reacted with Alexa Fluor 488‐NHS ester or Alexa Fluor 647 NHS ester (Click Chemistry Tools), respectively, for 2 h at 4 °C. Fluorochrome‐modified proteins were purified via spin filtration over 10 kDa Amicon Spin Filters (Sigma‐Aldrich) and washed 5 times with PBS. Concentration of purified proteins was determined by UV–vis absorbance measurements at 280 nm, after correcting for fluorophore absorbance, using the Nanodrop One (Thermo Fisher Scientific). O_2_‐Cryogels containing the fluorescently labeled RBD and N proteins were fabricated as described above. After thawing, cryogels were washed 4 times with 1 mL of PBS and imaged by confocal microscopy (Zeiss 800).

### Release of Immunomodulatory Factors and Antigens from Cryogels

To determine the in vitro release kinetics of GM‐CSF, CpG ODN 1826, and RBD from Cryogel_VAX_ and O_2_‐Cryogel_VAX_, gel samples were briefly washed in 70% ethanol followed by 2 PBS washes. Each washed gel was incubated in sterile PBS with 2% BSA in a microcentrifuge tube under orbital shaking at RT. The entire supernatant was removed periodically and replaced with the same amount of fresh buffer. GM‐CSF, CpG ODN 1826, and RBD released in the supernatant were detected by either ELISA (GM‐CSF: BioLegend ELISA MAX Deluxe, RBD: Elabscience SARS‐CoV‐2 Spike Protein S1 RBD ELISA Kit) or iQuant ssDNA quantification assay (GeneCopoeia, Inc.). The N protein release kinetics were not determined due to the instability of the protein under our experimental conditions.

### Antibody Titration by Enzyme‐Linked Immunosorbent Assay

Anti‐RBD IgG and IgM antibody titers were determined using a SARS‐CoV‐2 Spike S1‐RBD IgG and IgM ELISA detection kit (Genscript). Anti‐N IgG and IgM antibody titers were determined using a SARS‐CoV‐2 Nucleocapsid Protein IgG ELISA Kit (Lifeome). Both kits were optimized by replacing the HRP‐conjugated IgG or IgM anti‐human antibody with an HRP‐conjugated IgG (H + L) goat anti‐mouse antibody (Thermo Fisher Scientific) or an HRP‐conjugated IgM (heavy chain) goat anti‐mouse antibody (Thermo Fisher Scientific), respectively. Immunoglobulin isotyping was evaluated using Ig Isotyping Mouse Uncoated ELISA Kit (Thermo Fisher Scientific) and IgE Mouse ELISA kit (Thermo Fisher Scientific) following the manufacturer's recommendation by measuring absorbance at 450 nm on a plate reader (Synergy HT). All ELISAs were performed on mouse sera that were heat‐inactivated for 30 min at 56 °C. Endpoint titers were determined as the maximum dilution that emitted an optical density exceeding 4 times the background (i.e., sera from mice vaccinated with Sham vaccine).

### Severe Acute Respiratory Syndrome Coronavirus 2 Surrogate Virus Neutralization Test

The detection of neutralizing antibodies against SARS‐CoV‐2 that block the interaction between RBD and the human ACE2 (hACE2) cell surface receptor was determined using a sVNT according to the manufacturer's protocol (Genscript). Briefly, heat‐inactivated mouse sera were pre‐incubated with HRP‐RBD (30 min at 37 °C) to allow the specific binding of neutralizing antibodies. Then, the mixture was transferred into a plate coated with hACE2 and incubated for 15 min at 37 °C. The unbound HRP‐RBD, as well as, HRP‐RBD bound to non‐neutralizing antibody, will interact with the hACE2, while neutralizing antibody‐HRP‐RBD complexes will remain in suspension and will be removed during washing. TMB substrate was used to detect the non‐neutralized HRP‐RBD. Therefore, the absorbance was inversely proportional to the titer of anti‐SARS‐CoV‐2 neutralizing antibodies. For this experiment, tenfold dilutions of mouse sera (10^–1^ to 10^–8^) were used.

### Cytokine Quantification

Cytokine levels in mouse sera and cryogels were quantified using LEGENDplex mouse Th cytokine panel (BioLegend) according to the manufacturer's recommendations. Mouse sera were collected at day 24 (day 3 post‐boost) and diluted 10 and 100 times. Cryogel_VAX_, O_2_‐Cryogel_VAX,_ and (blank) cryogels were explanted at day 56, homogenized through a 70 µm cell strainer (Thermo Fisher Scientific), resuspended in 1 mL PBS, and then centrifuged 5 min at 300 × *g*. The supernatant was collected and diluted 2, 5, and 10 times. The cytokine panel included: IL‐2, IL‐4, IL‐5, IL‐6, IL‐9, IL‐10, IL‐13, IL‐17A, IL‐17F, IL‐22, IFN*γ*, and TNF*α*.

### Authentic Severe Acute Respiratory Syndrome Coronavirus 2 Plaque Reduction Neutralization Test

Heat inactivated mouse serum samples were serially diluted in PBS using twofold dilutions starting at 1:50. Dilutions were prepared in duplicate for each sample and plated in duplicate. Each dilution was incubated in a 5% CO_2_ incubator at 37 °C for 1 h with 1000 plaque‐forming units mL^−1^ (PFU mL^−1^) of SARS‐CoV‐2 (isolate USA‐WA1/2020, BEI). Controls included 1) Dulbecco's Modified Eagle Medium (Gibco) containing 2% FBS (Gibco) and 100X antibiotic‐antimycotic (Gibco) to a final concentration of 1X as a negative control; and 2) 1000 PFU mL^−1^ SARS‐CoV‐2 incubated with PBS as a positive control. Each dilution or control (200 µL) was added to two confluent monolayers of NR‐596 Vero E6 cells (ATCC) and incubated in a 5% CO_2_ incubator at 37 °C for 1 h. A gentle rocking was performed every 15 min to prevent monolayer drying. Cells were then overlaid with a 1:1 solution of 2.5% Avicel RC‐591 microcrystalline cellulose and carboxymethylcellulose sodium (DuPont Nutrition & Biosciences) and 2x Modified Eagle Medium (Temin's modification, Gibco) supplemented with 100X antibiotic‐antimycotic (Gibco) and 100X GlutaMAX (Gibco) both to a final concentration of 2X, and 10% FBS (Gibco). The plates were then incubated in a 5% CO_2_ incubator at 37 °C for 2 days. The monolayers were fixed with 10% neutral buffered formalin for at least 6 h (NBF, Sigma‐Aldrich) and stained with 0.2% aqueous Gentian Violet (RICCA Chemicals) in 10% NBF for 30 min, followed by rinsing and plaque counting. The half maximal inhibitory concentrations (IC_50_) were calculated using GraphPad Prism 8 as previously described.^[^ ^34^ ^]^

### Immune Cell Characterization in Cryogels and Lymph Nodes

At day 21 and 56, cryogels and LNs were explanted, homogenized over a cell strainer, and single cell suspensions were washed with PBS. Next, cells were stained with fixable viability dye eFluor 506 (eBioscience, 1:1000 dilution in PBS) for 30 min at 4 °C. The cells were subsequently washed once with PBS and twice with PBA (PBS + 1% BSA) before being stained overnight with fluorochrome‐conjugated antibodies (I‐A/I‐E‐FITC (Clone: M5/114.15.2), CD138‐PE (Clone 281–2), CD4‐PerCP‐Cy5.5 (Clone GK1.5), CD45.2‐PE‐Cy7 (Clone 104), CD11c‐APC (Clone N418), CD8‐AF700 (Clone 53–6.7), CD19‐APC‐Cy7 (Clone 6D5), CD11b‐BV421 (Clone: M1/70), CD3‐BV605 (Clone 145‐2C11), Biolegend) in PBA at 4 °C. Lastly, cells were washed 3 times with PBA, fixed in a 4% paraformaldehyde (PFA) solution (diluted in PBS) for 15 min at 4 °C, and then washed again 3 more times with PBA prior to analysis. Flow cytometry measurements were done using the Attune NxT flow cytometer (Thermo Fisher Scientific).

### Splenocyte Activation and Intracellular Cytokine Staining

Splenocytes were incubated with either 20 ng mL^−1^ phorbol myristate acetate (Sigma‐Aldrich) and 1 ug mL^−1^ ionomycin (Cell Signaling Technology), S protein‐derived peptides (GenScript), N protein‐derived peptides (GenScript), or control (no stimulation) in 1X Brefeldin A and 1X Monensin (Biolegend) solutions for 6 h at 37 °C. Next, the cells were washed with PBS and incubated for 30 min with Fixable Viability Dye eFluor 780 (eBioscience) in PBS (1:1000 dilution) at 4 °C. Cells were then washed once with PBS and twice with PBA before being stained overnight with fluorochrome‐conjugated antibodies (CD3‐FITC (Clone 145‐2C11), CD4‐PerCP‐Cy5.5 (Clone GK1.5), CD8‐AF700 (Clone 53–6.7), CD44‐BV605 (Clone IM7), Biolegend) in PBA at 4 °C. Cells were subsequently washed 3 times with PBA, fixed in 4% PFA, and then permeabilized using a Cyto‐Fast Fix/Perm Buffer Set (Biolegend) according to the manufacturer's protocol. Intracellular staining was performed by incubating the cells with fluorochrome‐conjugated antibodies (IL‐13‐PE (Clone: W17010B), IL‐4‐PE‐Cy7 (Clone: 11B11), IL‐17‐APC (Clone: TC11‐18H10.1), IL‐5‐BV421 (Clone: TRFK5), IFN*γ*‐BV510 (Clone XMG1.2), Biolegend) in permeabilization buffer for 30 min at 4 °C. Lastly, cells were washed 3 times with permeabilization buffer, resuspended in PBA, and analyzed using the Attune NxT flow cytometer (Thermo Fisher Scientific).

### Statistical Analysis

Flow cytometry data were processed using FlowJo software and manual gating was performed as depicted in Figures S4A and S5A, Supporting Information. Statistical analysis was conducted using GraphPad Prism 5 software. Data were analyzed using one‐way ANOVA and Bonferroni post‐tests to evaluate differences between time points (underlined dark stars indicate statistical significance) or two‐way ANOVA and Bonferroni post‐tests to evaluate the difference between different conditions/treatments (colored stars indicate statistical significance within a given condition of the same color). Values represent the mean ± standard error of the mean and *p*‐values below 0.05 were considered statistically significant.

## Conflict of Interest

The authors declare no conflict of interest.

## Author Contributions

T.C., L.J.E., and Z.J.R. contributed equally to this work. T.C., L.J.E., and S.A.B. conceived and designed the experiments. T.C., L.J.E., and Z.J.R. performed the experiments. T.C., L.J.E., Z.J.R., and S.A.B. analyzed the data and wrote the manuscript. T.C., L.J.E., Z.J.R., and S.A.B. conceived the figures. PRNT assay (Figure 2E) was designed by L.G.A.M., performed by L.G.A.M., L.E.A., R.I.J., and N.S., and analyzed by T.C., R.I.J., and N.S. All authors discussed the results, commented on, and proofread the manuscript. The principal investigator is S.A.B.

## Supporting information

Supporting Information

## Data Availability

The data that support the findings of this study are available from the corresponding author upon reasonable request.
