# Supplementary material for: Biomaterials and Oxygen Join Forces to Shape the Immune Response and Boost COVID‐19 Vaccines
Source: Adv Sci (Weinh). 2021 Jul 20;8(18):2100316. doi: 10.1002/advs.202100316 (PMC8209904; doi:10.1002/advs.202100316)
Supplement: Supplementary file 1 — Supporting Information [file ADVS-8-2100316-s001.pdf]

© 2021 Wiley-VCH GmbH

ADVANCED SCIENCE
Open Access

Supporting Information

for *Adv. Sci.*, DOI: 10.1002/advs.202100316

Biomaterials and oxygen join forces to shape the immune response and boost COVID-19 vaccines

*Thibault Colombani, Loek J. Eggermont, Zachary J. Rogers, Lindsay G. A. McKay, Laura E. Avena, Rebecca I. Johnson, Nadia Storm, Anthony Griffiths, Sidi A. Bencherif**

Supporting Information

Biomaterials and oxygen join forces to shape the immune response and boost COVID-19 vaccines

Thibault Colombani[1‡], Loek J. Eggermont[1‡], Zachary J. Rogers[1‡], Lindsay G. A. McKay[2], Laura E. Avena[2], Rebecca I. Johnson[2], Nadia Storm[2], Anthony Griffiths[2], Sidi A. Bencherif[1,3,4,5*]

[1] Department of Chemical Engineering, Northeastern University, Boston, MA 02115, USA.

[2] Department of Microbiology and National Emerging Infectious Diseases Laboratories, Boston University School of Medicine, Boston, MA 02118, USA

[3] Department of Bioengineering, Northeastern University, Boston, MA 02115, USA.

[4] Harvard John A. Paulson School of Engineering and Applied Sciences, Harvard University, Cambridge, MA 02138, USA.

[5] Biomechanics and Bioengineering (BMBI), UTC CNRS UMR 7338, University of Technology of Compiègne, Sorbonne University, 60203 Compiègne, France.

‡Authors contributed equally

* Corresponding author: s.bencherif@northeastern.edu

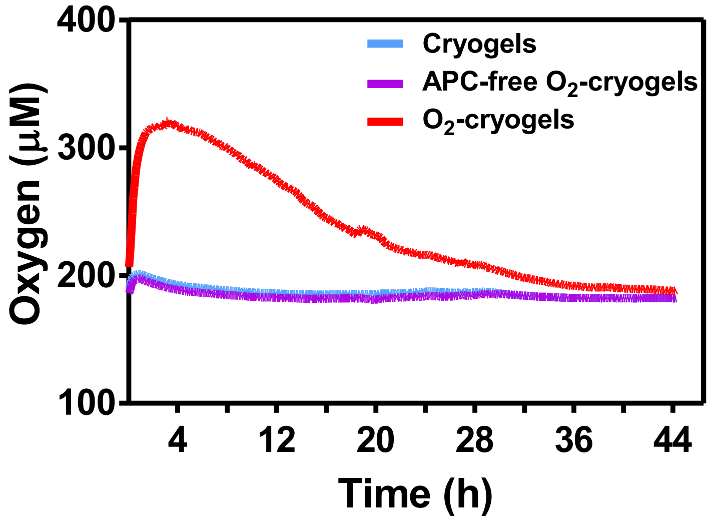

Figure S1. O_2-cryogels enable controlled release of oxygen in their surrounding environment. Oxygen release from Cryogels, APC-free (no catalase) O_2-cryogels, and (catalase containing) O_2-cryogels under normoxic conditions. Data are representative of n=4 experiments.

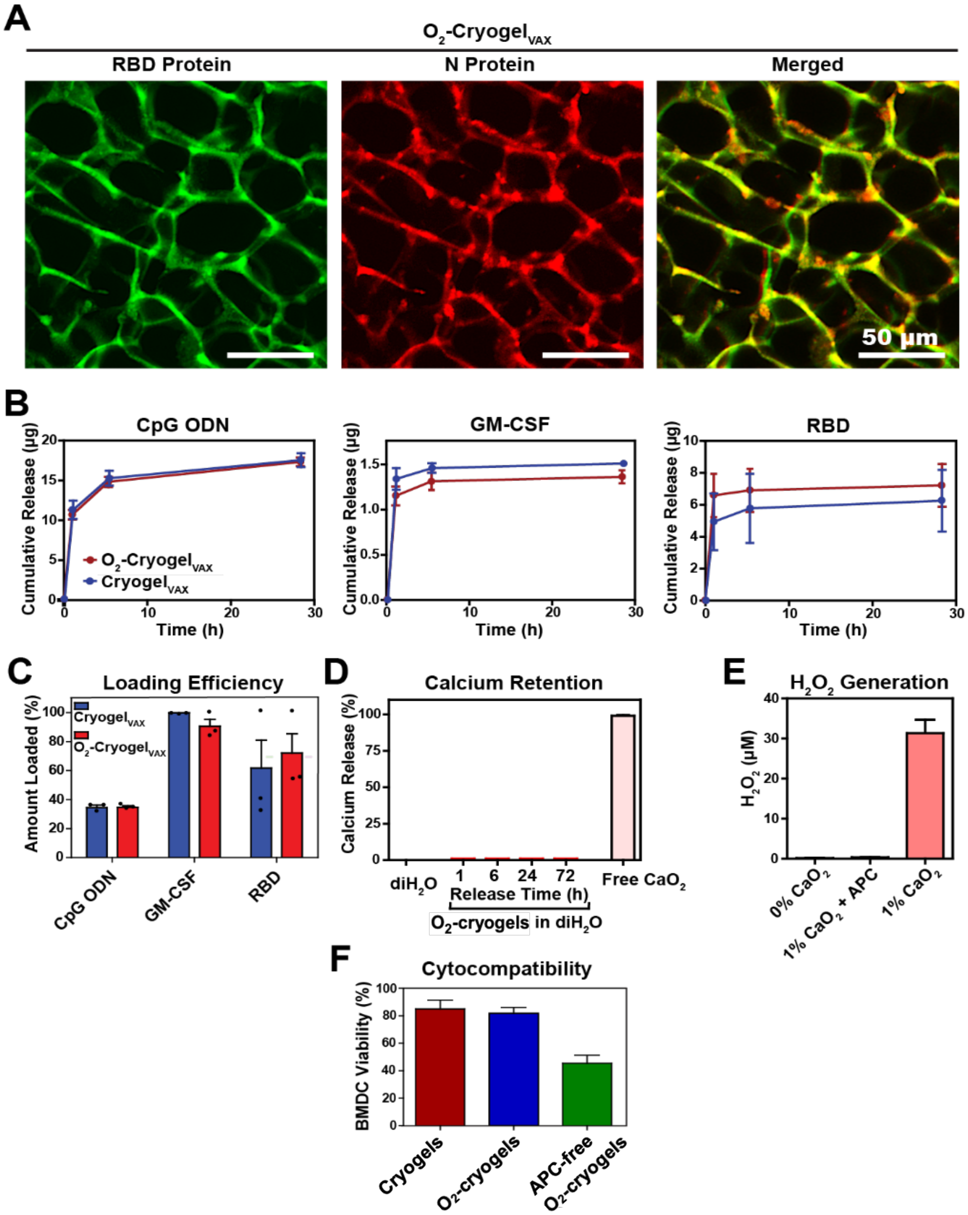

Figure S2. Efficient encapsulation and sustained release of SARS-CoV-2 subunit proteins and immunomodulatory factors from cryogels. (A) Confocal microscopy images showing co-encapsulation of Alexa Fluor 488-labeled RBD and Alexa Fluor 647-labeled N protein within the polymer walls of O_2-Cryogel$_{VAX}$. (B) Cumulative release and (C) Loading efficiency of CpG-ODN 1826, GM-CSF and RBD in Cryogel$_{VAX}$ and O_2-Cryogel$_{VAX}$. (D) Calcium release from O_2-cryogels, incubated at 37°C in diH_2O, and quantified using a hardness test. (E) H_2O_2 release from cryogels (no CaO_2), O_2-cryogels (1% CaO_2 + APC) and APC-free O_2-cryogels (1% CaO_2, no APC). (F) BMDC viability after 24 h of incubation within Cryogels, O_2-cryogels, and APC-free O_2-cryogels under normoxic conditions. Values represent the mean ± SEM (n=3–5). Data were analyzed using two-way ANOVA and Bonferroni post-tests to evaluate the difference between different conditions/treatments at the same time point (colored stars indicate statistical significance within a given condition of the same color), $*p < 0.05$.

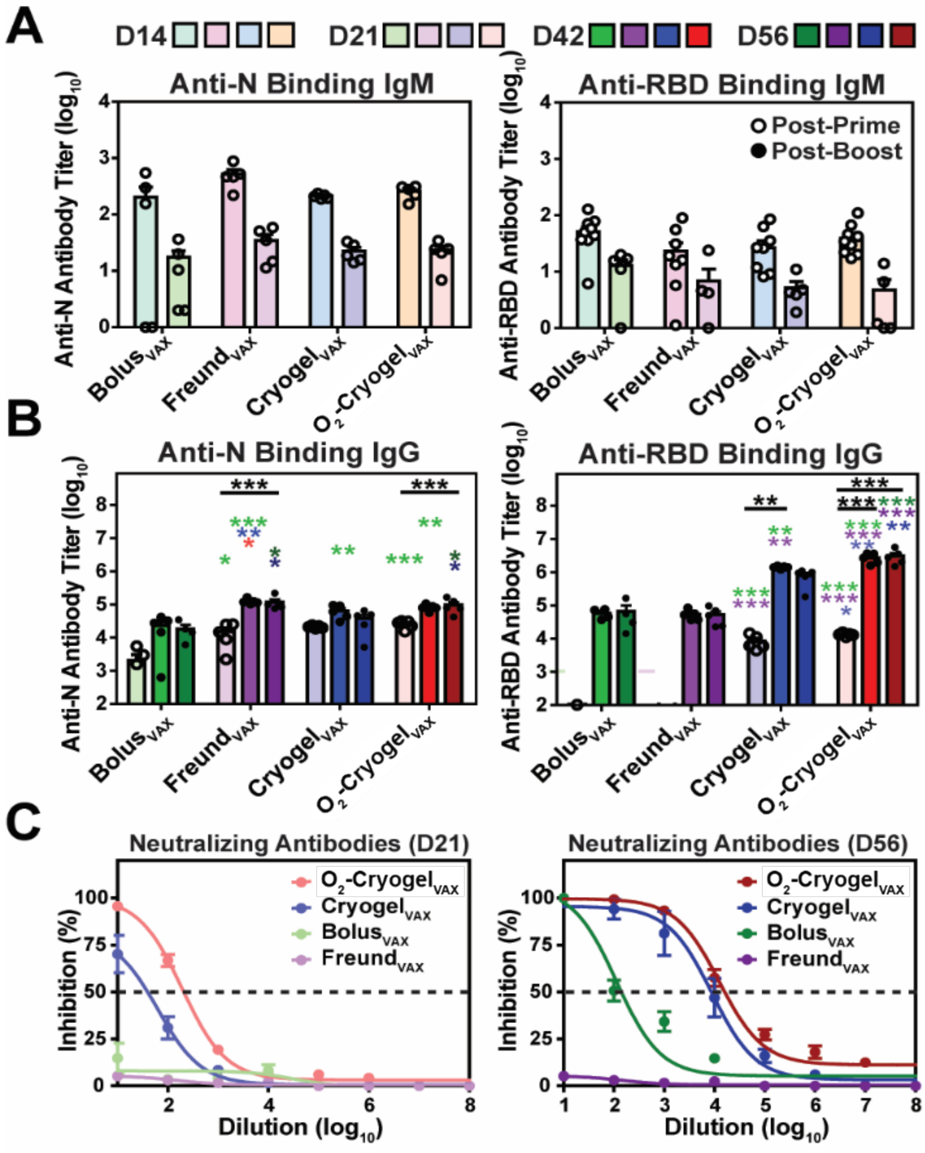

Figure S3. O_2-Cryogel$_{VAX}$ enhances the humoral immune response against SARS-CoV-2. (A–B) Endpoint titers of RBD- and N- specific IgM (A: day 14 and 21) and IgG (B: day 21, 42 and 56) antibody determined by ELISA. (C) Inhibition rate as the function of serum dilution of the SARS-CoV-2 surrogate virus neutralization test at day 21 and 56. Values represent the mean ± SEM (n=5). Data were analyzed using one-way ANOVA and Bonferroni post-tests to evaluate differences between time points (underlined dark stars indicate statistical significance) or two-way ANOVA and Bonferroni post-tests to evaluate differences between conditions at the same time point (colored stars indicate statistical significance within a given condition of the same color), $*p < 0.05$, $**p < 0.01$ and $***p < 0.001$.

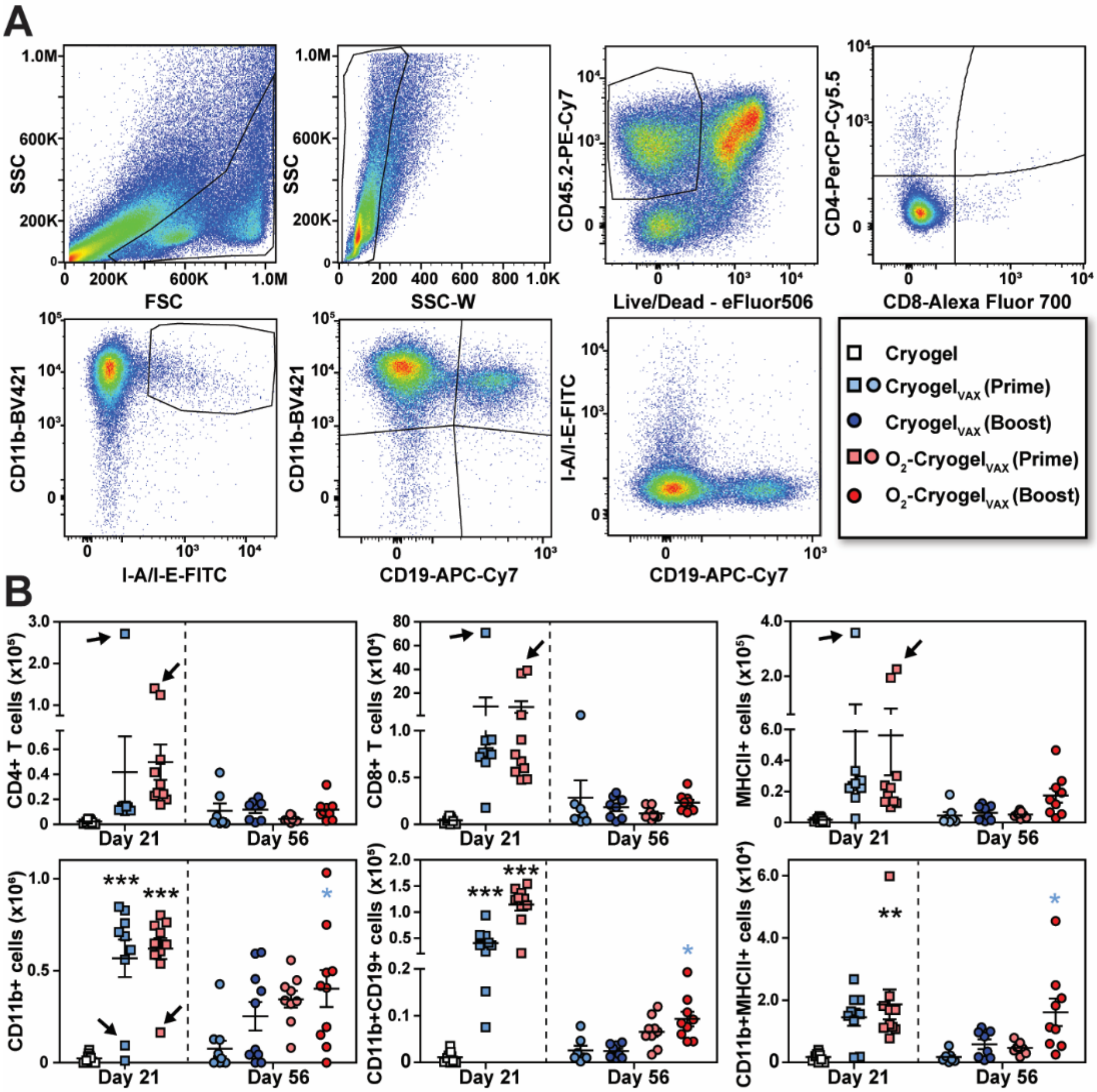

Figure S4. Cryogels contain limited numbers of adaptive immune cells after 56 days. (A) Flow cytometry gating strategy and (B) cell numbers in cryogels at day 21 and 56 post-vaccination. Arrows indicate corresponding gels with high numbers of T cells and MHCII+ B cells. Values represent the mean ± SEM (n=5). Data were analyzed using two-way ANOVA and Bonferroni post-tests to evaluate differences between conditions at the same time point (colored stars indicate statistical significance within a given condition of the same color), $*p < 0.05$, $**p < 0.01$ and $***p < 0.001$.

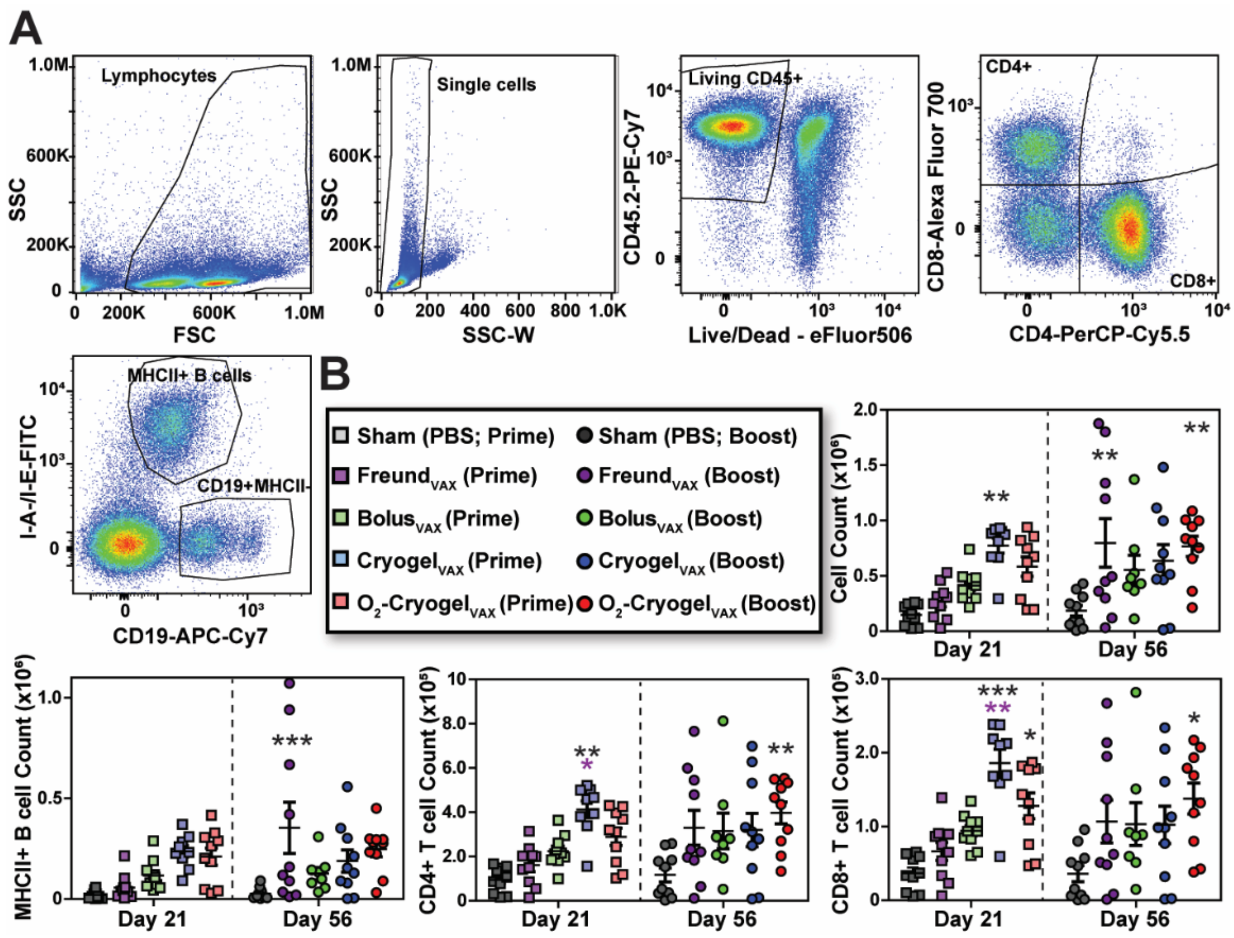

Figure S5. Vaccination with Cryogel$_{VAX}$ and O_2-Cryogel$_{VAX}$ increases adaptive immune cell numbers in draining LNs. (A) Flow cytometry gating strategy and (B) cell numbers in LNs at day 21 and 56 post-vaccination. Two draining LNs were analyzed per animal. Values represent the mean ± SEM (n=5). Data were analyzed using two-way ANOVA and Bonferroni post-tests to evaluate differences between conditions at the same time point (colored stars indicate statistical significance within a given condition of the same color), *$p < 0.05$, **$p < 0.01$ and ***$p < 0.001$.

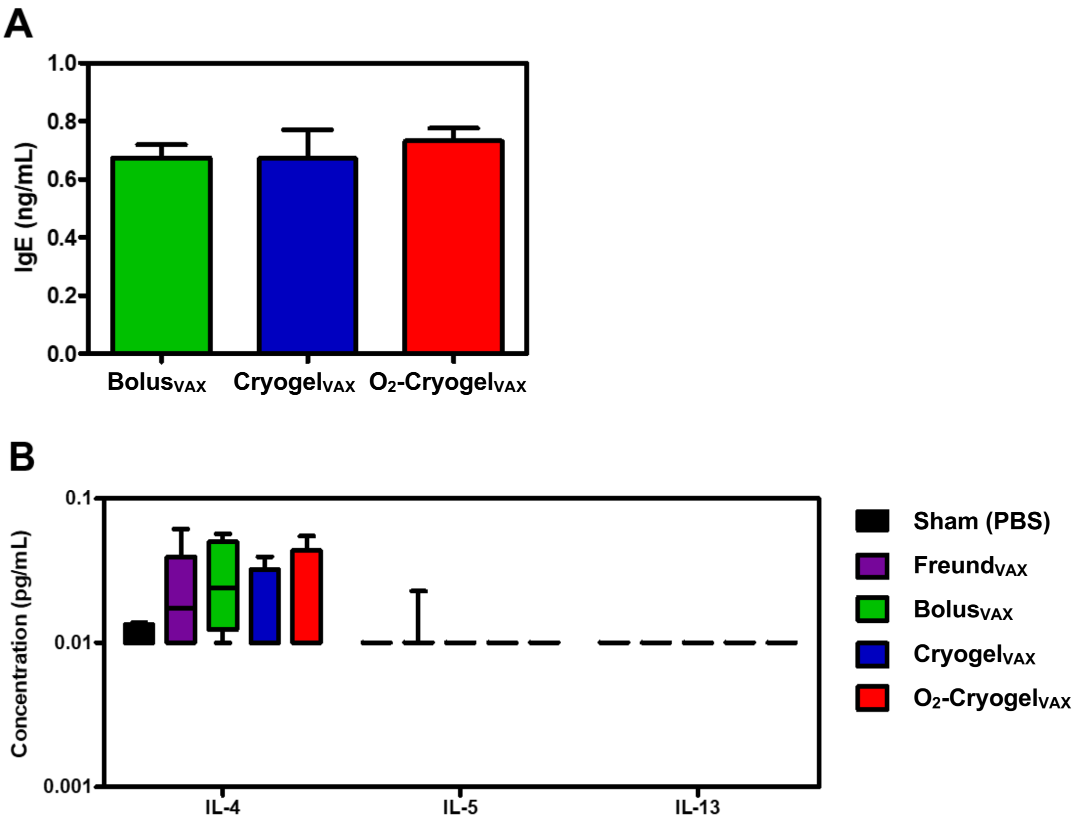

Figure S6. Immunization with O_2-Cryogel$_{VAX}$ does not trigger an allergic response. (A) Mouse serum IgE titers were assessed by ELISA at day 56 across all the vaccine conditions. (B) Mouse serum IL-4, IL-5, IL-13 levels were measured at day 24 with a multiplex immunoassay across all the vaccine conditions. Values represent the mean ± SEM (n=5).

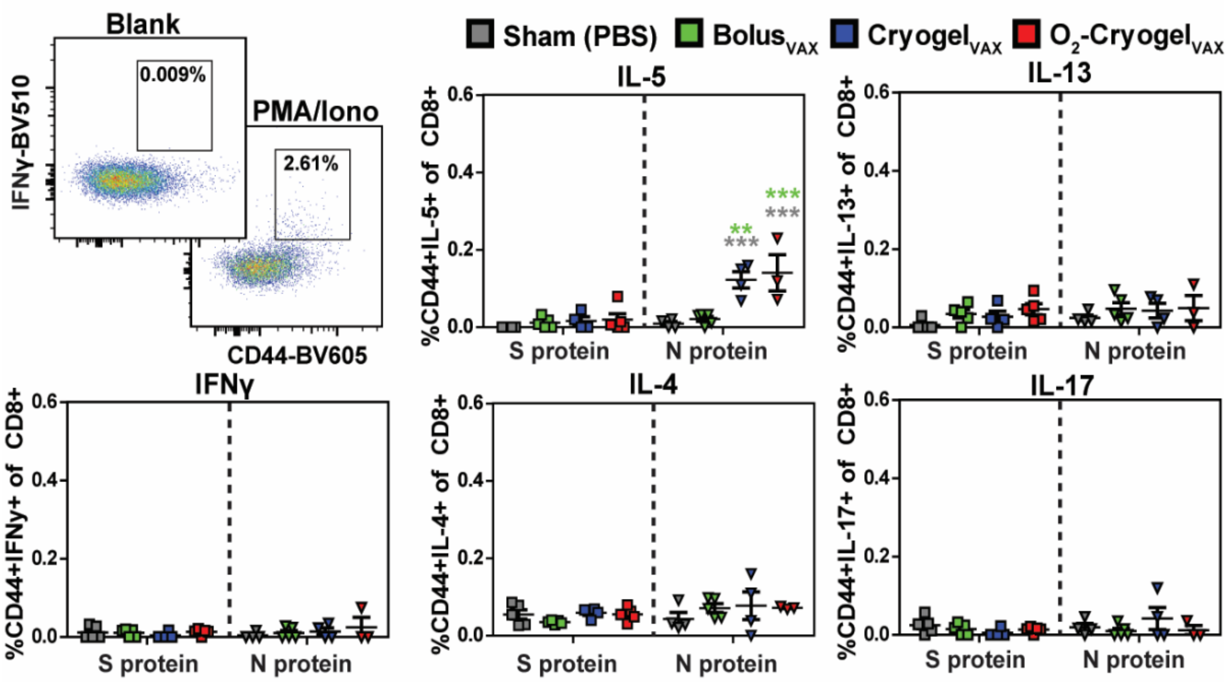

Figure S7. O$_2$-Cryogel$_{VAX}$ induces IL-5-producing N-specific CD8+ T cells. Flow cytometry gating and frequencies of cytokine-producing CD44+CD8+ T cells following S and N protein-derived peptide stimulation of splenocytes isolated at day 21. Values represent the mean ± SEM (n=5). Data were analyzed using two-way ANOVA and Bonferroni post-tests to evaluate the difference between conditions/treatments at the same time point (colored stars indicate statistical significance within a given condition of the same color), *$p < 0.05$, **$p < 0.01$ and ***$p < 0.001$.
